# Crimean-Congo Haemorrhagic Fever in Kosova : a fatal case report

**DOI:** 10.1186/1743-422X-3-85

**Published:** 2006-10-12

**Authors:** Salih Ahmeti, Lul Raka

**Affiliations:** 1 Infectious Disease Clinic, University Clinical Centre of Kosova & Faculty of Medicine, Prishtina University, Rrethi i spitalit, p.n., 10 000 Prishtina, Kosova; 2 Department of Microbiology, National Institute of Public Health of Kosova & Faculty of Medicine, Prishtina University, Rrethi ispitalit, p.n., 10 000 Prishtina, Kosova

## Abstract

Crimean-Congo haemorrhagic fever (CCHF) is an often fatal viral infection described in about 30 countries around the world. The authors report a fatal case of Crimean-Congo hemorrhagic fever (CCHF) observed in a patient from Kosova. The diagnosis of CCHF was confirmed by reverse transcription-PCR. Late diagnosis decreased the efficacy of treatment and patient died due to severe complications of infection.

## Background

Crimean-Congo haemorrhagic fever (CCHF) is a tick-born disease caused by a *Nairovirus *of the Family *Bunyaviridae*. Infection is transmitted to humans by *Hyalomma *ticks or by direct contact with the blood or tissues of infected humans or viraemic livestock [1,2]. Clinical features usually include a rapid progression characterised by haemorrhage, myalgia and fever, with a mortality rate of up to 30%. CCHF virus has a wide geographic distribution, circulating in Africa, the Middle East, Asia, and Central and South-Eastern Europe [3]. CCHF was first clinically described in 1944 in Crimea in the former Soviet Union during a large outbreak of over 200 cases [4]. CCHF virus was identified in 1967, from a patient in Uzbekistan, and was found to be similar to a virus isolated in 1956 in Congo, hence the name Crimean-Congo [5,6].

The Balkan Peninsula is an endemic region for the disease, sporadic cases or even outbreaks being observed every year. The first case in Kosova occurred in 1954 [7]. There were no registered cases of CCHF in Kosova until 1989(7 cases). Three outbreaks occurred in 1995, 2001 and 2004 with, overall, 186 serologically confirmed cases of the disease, with a case fatality rate of 27% [8]. There were also a large number of patients who presented with clinical features of haemorrhagic fever, but confirmatory serological diagnosis was not available due to technical reasons. Kosova is also known as an endemic region for hemorrhagic fever with renal syndrome (HFRS) caused by hantaviruses, which can coexist with CCHF viruses.

## Case presentation

A previously healthy 8-year-old boy, living in the village Carallukë near Prishtina, Kosova, at the end of May 2004, was caring for livestock in the meadow and bathing in a nearby stream for three consecutive days. His father recalled that the boy had removed a tick from his head about five days before the onset of the disease.

The disease started on 27 May, with chills, myalgia, cough, nausea, anorexia, vomiting, headache and backache. On 28 May, the patient visited the family doctor in an outpatient clinic and received ambulatory care (antibiotics, corticosteroids and antipyretics). On May 29, he visited a pediatrician in a private outpatient clinic in the regional health care centre in Prizren, where broad-range antibiotic therapy was initiated. The doctor recommended hospitalization in case of non-response to therapy, since the patient came from an area in which CCHF was endemic.

On 31 May, the patient was admitted to the Pediatric Clinic at the University Clinical Centre of Kosova, the only tertiary care center for an estimated 2.1 million inhabitants of Kosova. On admission to the clinic, the patient presented with chills, cough, vomiting, headache, backache and pain in both legs.

On initial examination, his vital signs included a body temperature of 40°C, a pulse of 106 beats/min and a respiratory rate of 40 breaths/min. The patient was orientated, without neurological symptoms, but prostrate.

The patient was anaemic with an erythrocyte count and hemoglobin level of 3.4 × 10^12 ^cells/litre (normal range, 4.5 × 10^12 ^- 5.9 × 10^12 ^cells/litre) and 11.5 g/dl (normal range, 13.5 – 17.5 g/dl), respectively. Thrombocytopenia was noted, with a platelet count of 59.2 × 10^9^/litre (normal range, 140 × 10^9 ^- 400 × 10^9^/litre). Mild hyperbilirubinemia, hypoproteinemia, and hypoalbuminemia were also present. The patient was treated with intravenous antibiotics, corticosteroids and antipyretics according to presenting sepsis syndrome and lack of haemorrhagic syndrome in admission to tertiary care center. However, the symptoms did not improve and the patient was not recovering. Moreover, despite a decrease in temperature to 38°C on 2 June, the boy developed epistaxis and gingival bleeding. He was anxious and this was accompanied by uncontrolled screaming. Next day, he was transferred to the Infectious Disease Clinic in a serious condition, not fully conscious, and with diffuse haemorrhagic signs appearing, with purpuric lesions on the trunk and face, and with gingival bleeding. Large ecchymoses appeared at the sites of venepuncture.

The heart was in sinus rhythm: 110 beats/min with weak pulse tone. The abdomen was tender on palpation and the liver and spleen were both palpable. Meningeal signs were slightly positive. Abdominal ultrasonography revealed hepatosplenomegaly and the presence of free liquid in the abdominal cavity, suggestive of hemoperitoneum. Fifty ml of blood was withdrawn from the abdominal cavity by paracentesis. Lumbar puncture revealed clear cerebrospinal fluid without increase in cellular elements.

On 4 and 5 June, the patient developed massive hemorrhage with hematemesis, melena, and petechiae. Epistaxis continued and nasal tamponade was undertaken.

The erythrocyte count and hemoglobin level decreased from 3.4 to 2.9 × 10^12 ^cells/litre and from 11.5 to 10.8 g/dl, respectively. An infusion of fresh platelets partially corrected the thrombocytopenia, and the count increased from 59.2 to 96.3, but this later decreased again to 64 × 10^9^/litre.

The alanine transaminase and lactate dehydrogenase levels were 164 U/litre (normal range, 5 to 40 U/litre) and 287 U/liter (normal range, 114 to 240 U/litre), respectively, suggesting liver dysfunction. Activated partial thromboplastin time was 110 seconds(= 60 seconds) and the fibrinogen level was 80 mg/dL(normal value = 110 mg/dL). Coagulation factors (II, V, VII, X) were decreased. Serological tests for hepatitis were negative.

Supportive therapy given to the patient during the course of the disease consisted of hydration, antibiotics and control of temperature. Blood transfusions, two plasma and three platelets solutions were administered to the patient. Ribavirin was not administered because it was not available and the patient had already had a week with symptoms. Despite the treatment, the clinical features deteriorated and the patient died on 6 June due to haemorrhagic shock and pulmonary oedema.

The blood samples drawn on 3 June for serological and molecular testing were referred to the WHO Collaborating Centre for Arbovirus and Haemorrhagic Fever Reference and Research in Ljubljana, Slovenia. This centre provides laboratory support for the CCHF in Kosova. ELISA tests for CCHF and Hantan-virus were negative in the serum sample, whereas the diagnosis of CCHF was confirmed by reverse transcription-PCR from serum and blood obtained during paracentesis.

Complete S segment of the Kosovo Hoti strain was confirmed in Slovenia [9]. It was deposited under the [GenBank : DQ133507]. Phylogenetic studies have shown that the Kosovan strain is grouped together in the clade with the Southwest Russian and Turkish strains and is phylogenetically most closely related to Drosdov strain of CCHFV [10].

## Conclusion

From this report, the most important lesson to be derived is that late diagnosis decreases the efficacy of treatment and aggravates the outcome of the disease. Diagnosis of CCHF is important to prevent the spread of CCHF virus among the health-care workers and relatives of patients. Treatment with ribavirin may be useful if given within the early stage of disease [11]. The presence of visceral bleeding is a predictor of poor prognosis. The other lesson to be learned from this case is that every febrile haemorrhagic syndrome encountered in endemic areas, such as parts of Kosova, should probably be considered to be viral haemorrhagic fever, until proven otherwise.

## Competing interests

The author(s) declare that they have no competing interests

## Authors' contributions

SA participated in acquisition, analysis and interpretation of data. LR participated in the design of the study and drafted the manuscript. Both authors read and approved the final manuscript.

**Figure 1:**
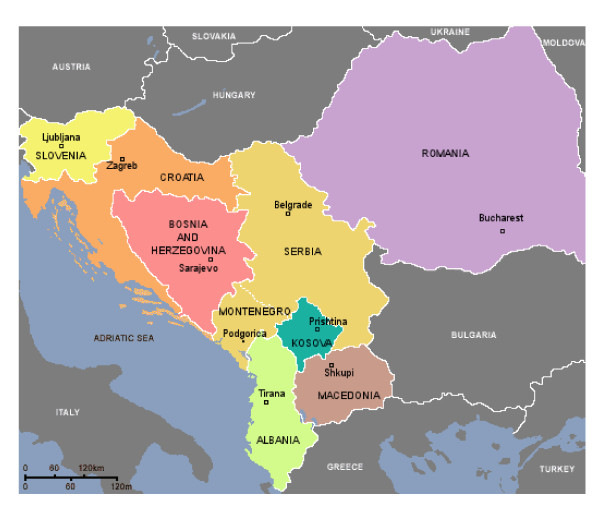
Map of the South-East Europe.
